# Assessment of Accumulation Processes at the Middle Pleistocene Site of Ambrona (Soria, Spain). Density and Orientation Patterns in Spatial Datasets Derived from Excavations Conducted from the 1960s to the Present

**DOI:** 10.1371/journal.pone.0167595

**Published:** 2016-12-21

**Authors:** Laura Sánchez-Romero, Alfonso Benito-Calvo, Alfredo Pérez-González, Manuel Santonja

**Affiliations:** 1 Centro Nacional de Investigación sobre la Evolución Humana (CENIEH), Paseo Sierra de Atapuerca 3, Burgos, Spain; 2 Escuela Interuniversitaria de Posgrado en Evolución Humana, Universidad de Burgos, Juan de Austria 1, Burgos, Spain; Max Planck Institute for the Science of Human History, GERMANY

## Abstract

The Middle Pleistocene site of Ambrona (Soria, Spain) is a major reference for European Acheulean studies. The origin of the lithic and fauna accumulations at this site was first thought to be anthropogenic, but later studies showed that it was mainly natural. The first person to conduct excavations at the Ambrona site was the Marquis of Cerralbo, in 1914; other research groups followed in more recent times (the Howell & Freeman team and the Santonja & Pérez-González team). The digs yielded a great amount of information, but until now it had never been unified. In this paper, we compile all the available published and unpublished excavation documentation from the 1960s to the present. We use these maps and sections to present our spatial study of the LSM (Lower Stratigraphic Member) at the Ambrona site, combining stratigraphic criteria with GIS density and orientation analysis. This study enabled us to define the main concentrations of the LSM, providing an initial contribution to an assessment of their accumulation processes. Most of the concentrations preserved in the ancient shore area of the site display marked orientation patterns which coincide with the direction of the main water flows into the Ambrona wetland. However, random orientation patterns were observed in the central part of the site (Alpha concentration); they may be mostly preserved without undergoing transport processes, as previous taphonomic studies also confirm.

## Introduction

Site formation processes [1–4] are one of the main subjects investigated in Paleolithic archeology. In recent decades, archeologists have become more aware of the fact that the record of Palaeolithic sites was altered by natural agents such as slope dynamics, erosion, deflation, landslides, water flows and/or bioturbation [5–7]. Changes caused by animals must also be taken into account, especially at sites where there are evident signs of elephant presence. These animals can greatly modify the landscape on both a large and a small scale [8]. In Africa, they dig holes to reach underground water [9–11] and into deposits to ingest mineral-rich sediments [12]. In places where elephants congregate in large groups, such as favorite feeding patches or around water sources, their dung literally carpets large areas [8]. In varying degrees, almost every Paleolithic site in Europe has at least one disrupted context [5].

The archeological record in open-air sites is best preserved where the environment is particularly favorable; that is, where sedimentation is rapid and any remains are buried quickly [13]. Since they are not exposed for a long period of time, these materials are protected from alteration. Both lacustrine and palustrine environments are favorable for the preservation of remains, and sites located in such environments are usually considered examples of well-preserved archaeological assemblages [14–15]. Moreover, these types of environment, being permanently or seasonally saturated with water, attract animals seeking natural resources.

However, these areas undergo processes which can alter the original position of remains and their state of preservation. This is especially true in lakeside areas, where hominins were active and where lake-level changes, littoral processes and fluvial events could generate significant perturbations. Careful assessment of these processes is thus crucial in interpreting sites that originated in these environments.

In this paper, we analyze the effects of these processes at the Ambrona site, where two main environments have been identified: 1) two streams that flowed from NE and E into the wetland and left mostly cross-bedded gravels and sands in the stratigraphic sequence; and 2) a wetland area where low-energy conditions and rapid sedimentation of clays and marls may have generated more favorable conditions for the undisturbed preservation of archeological remains.

Other sites similar to Ambrona and containing well-preserved megafaunal remains include Áridos 1 and 2, Kärlich-Seeufer, Neumark-Nord 1 (NN1), La Polledrara and Castel di Guido.

Áridos 1 and 2, in Spain, are located on a floodplain (overbank facies) of the Jarama river and contain lithic artifacts spatially associated with partial elephant carcasses [16]. Áridos 1 yielded part of an adult female *Palaeoloxodon antiquus* skeleton; the remains rest on a consolidated paleosurface and were covered by overbank facies sediments [17]. The lithic industry was scattered in the immediate vicinity of the elephant remains. At Áridos 2, a *Palaeoloxodon antiquus*’s remains were found in anatomical connection. They lay on an ancient floodplain in muddy overbank deposits and in pebble and sandy low-energy channels. Human intervention was attested to by 34 lithic items associated with the elephant bones and by the presence of cut-marks on the scapula and on a rib [17–18].

The Kärlich-Seeufer site, in Germany, contains Acheulean artifacts and faunal remains dominated by *Palaeoloxodon antiquus*. This site used to be interpreted as an elephant-hunters’ camp, but several studies have since shown that the human activity took place in the vicinity of a small lake with prevailing oligotrophic conditions. The sediments and finds were deposited on the edge of and in a former swamp exposed at the Kärlich clay pit [19]. At NN1, also in Germany, elephant bones have been found in lacustrine deposits [20–22]. These consist of sediments deposited in the lake during successive episodes of rising and lowering of the lake level [23]. Elephant remains were found in lakeside sediments that emerged when the lake level dropped, and inside the lake itself, usually not far from the shore. The edges of lakes and ponds are excellent environments for the preservation of assemblages of vertebrate fossils. Moreover, the presence of swampy/muddy areas may also have led to rapid burial of the remains [24].

In Italy, the fluvial and fluvio-palustrine deposits of La Polledrara di Cecanibbio are similar to the fluvial deposits we found at Ambrona. Vertebrate remains are embedded in sandy and muddy sediments deposited in short-lived fluvial, palustrine and marshy environments [25]. The alternation of phases of flooding and of low water levels separated by periods with normal water flow produced erosive and depositional phenomena that shifted the position of bone remains [26]. Castel di Guido, likewise in Italy, is an Acheulean site about 20 km W-NW of Rome [27]. The industry and large bones are for the most part in their original position, whereas some of the remains were reworked in periods when stream flow resumed, and were carried at varying distances from their original location [28]. Geological and taphonomic studies suggest that the site is a palimpsest of several phases of frequentation by humans, with partial reworking and removal of the fine fraction by low-energy flow [29–31].

These studies show how important it is to make detailed stratigraphic, sedimentological and taphonomic analyses in order to further our understanding of accumulation processes in these environments. Several spatial methodologies have recently been proposed to gain valuable information on how natural processes affect the accumulation of remains. Fabric analysis applied to archeology is the study of the orientation of each find in an archeological deposit, by measuring its direction (bearing) and inclination (plunge); it is being used successfully to detect preferred orientations and assess natural perturbations [8], [32–36]. However, we do not have bearing and plunge measurements for all the Ambrona remains we are studying. Their absence has been partly offset with excavation maps, which have proved to be useful for accurately estimating the axial orientation of the archeological remains with the aid of Geographic Information Systems [28], [35], [37]. Moreover, orientation analysis based on excavation maps has been combined with hydrological models, such as flume models [38] and GIS flow accumulation models [39], providing interesting new insights in evaluating accumulation processes at Paleolithic sites.

This paper presents the density and orientation analysis of the remains mapped at the Ambrona site, combined with a detailed description of the stratigraphic and sedimentological context. To carry out this analysis, it was essential to compile an intensive dataset. The Ambrona site has been excavated over many years, in multiple phases and by different teams; therefore, at present there exist several different spatial datasets organized according to different coordinate systems. We compiled and georeferenced all the available published and unpublished spatial datasets, thereby obtaining an unprecedented full view of this site. In this integrated approach, we measured the orientation of the Ambrona remains by comparing several GIS methods [28], [37]. When orientation patterns vary across the site, the spatial variability of the orientations is usually estimated with the aid of an artificial grid [28], [39].

We studied this spatial orientation variability using a new method based on previous density mapping. Density analysis, a common method of data representation in archeology [40], enables easy graphic detection of areas where major accumulations of remains are located. Density values are usually calculated using a grid that divides the area being analyzed, and plotting the objects situated around each grid cell. This kind of study is used for small-scale intra-site interpretation of spatial patterns [41], which are influenced by the succession of different depositional contexts with different spatial distributions [42]. In our study, we applied the density method to assess the natural concentrations of items, and then made an orientation analysis for each natural concentration. This spatial study provides a basis for further functional interpretation of the Ambrona site in a comprehensive perspective.

## The Ambrona site

**Fig 1:**
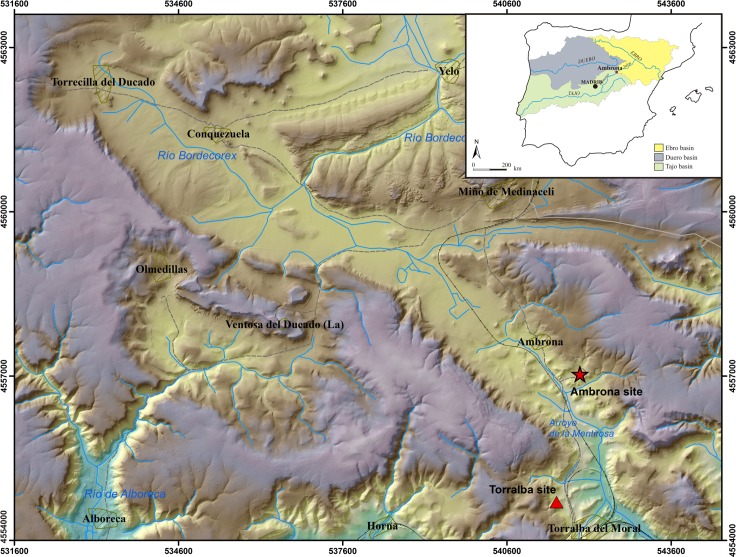
Location of the Pleistocene sites of Ambrona and Torralba in the polje of Conquezuela, in the south of the Soria province.

**Fig 2:**
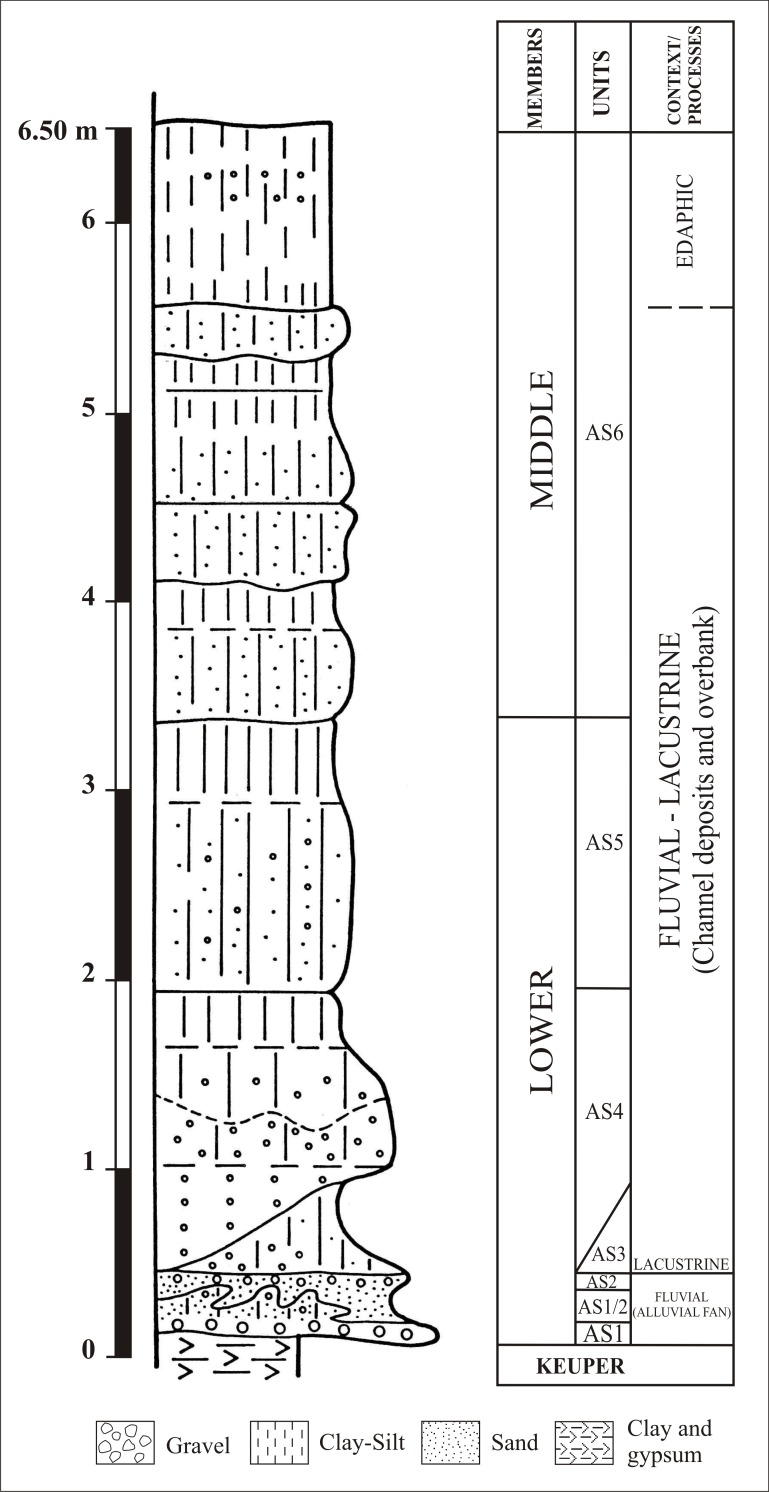
Lithostratigraphic sequence of the Ambrona site, defined by the S&PG team (after Pérez-González et al., 2005).

The Pleistocene site of Ambrona is located in the south of the Soria province (**Fig 1**), in the watershed area separating the drainage basins of the Ebro, Duero and Tagus rivers. This is a strategic corridor between the Meseta highlands and the Jalón valley, which herds of herbivores used in their seasonal migrations [43]. From the geological standpoint, Ambrona is located in the northern half of the Castilian Branch of the Iberian Range, near the continental Almazán basin (Tertiary), in a karstic *polje*-type context. During the Lower-Middle Pleistocene, the polje was already an open valley, 1 km wide and 12–13 km long, that ran from north-west to south-east. In the second half of the Middle Pleistocene, a wetland area developed on the flat and impermeable valley floor, and sediments containing faunal and Acheulean lithic remains were deposited. The environment was lacustrine and fluvial, with alluvial fans (**Fig 2**) [44]. A horse tooth found in the level AS6 sequence was dated through combined ESR/U-series analysis, thus establishing that the Middle Unit of Ambrona is about 350 ka old (MIS 10). The Lower Unit would have been deposited just before the middle one, probably during the MIS 11 [45].

The archeological investigation of the Ambrona Paleolithic site started in 1914, on the initiative of Enrique de Aguilera y Gamboa, Marquis of Cerralbo (1845–1922). In 1962–1963, 1980, 1981 and 1983, an international team led by F. Clark Howell and L. G. Freeman (hereinafter the H&F team) carried out extensive excavations and investigations at the site, excavating 2,700 m^2^ of deposit. Howell’s technique was based on the Wheeler method [46], digging trenches and interpreting stratigraphic layers. The area of the grid squares was 9 m^2^ and the sequence was excavated down to the Keuper Formation; the walls of the squares made it possible to observe the continuity and changes in the stratigraphy. The two lithostratigraphic units identified [47–48] were named Lower and Upper Member Complexes. Howell et al. (1995) described the “Torralba Formation” at Ambrona as comprising a Lower Member Complex, consisting of six levels (IIa to IVb), and an Upper Member Complex, consisting of four levels (Va to Vd) [47]. This interpretation has been rejected by the current team, which noted that the stratigraphies of Ambrona and Torralba are not correlated [44], [48].

Excavated remains were left on the surface they were found on. After the deposit was fully described, the walls of the archeological grid squares were knocked down to enable an overall view of the exposed materials. The items were then recorded on a map, thereby producing an exhaustive record of the finds. This work enabled us to identify the large accumulations that the H&F team had found at Ambrona; the stratigraphic units they are attributed to are the ones defined at that time: the Lower and Upper Member Complexes. However, only a few of the spatial datasets collected in these excavations were ever published: only three studies mention the spatial distribution maps of the remains [49–51] and one of them was used as a text background image.

More recently, from 1993 to 2000, a total of 688 m^2^ were excavated by Santonja and Pérez-González and their collaborators (hereinafter the S&PG team) [52–53]. The S&PG team focused on the features of the different lithostratigraphic units and their levels. The goals were to understand how they were formed, and propose working hypotheses regarding the different levels excavated, to analyze the chronostratigraphic context and to integrate the data that had not been interpreted before. During these years an exhaustive record was created: 400 linear meters of detailed stratigraphies, drawings of the excavated materials, control point surveys with a total station, and maps.

Many species have been identified in the faunal assemblages of Ambrona [48], [54]. They are grouped in two large consecutive units, one characterized by the presence of megafaunal remains, especially of *Palaeoloxodon antiquus*, and Acheulean lithic industry, the other by the presence of *Equus* remains and Middle-Paleolithic lithic industry. Ambrona’s faunal association is typical of the late (but not final) Middle Pleistocene. The list of mammals found in the Lower Unit suggests an environment consisting of forests, open grasslands and areas with water resources, where the climate was temperate and rather humid. Paleopollens and biomineralizations (phytoliths) have likewise provided information about the kind of environment the deposits of Ambrona were formed in [55–57]. Pollen analysis indicates a milder climate, comparable to today’s. Herpetofaunal remains confirm these interpretations and indicate that the climate at the time was similar to the one at Ambrona today, though summers were not so dry then, and winter temperatures were slightly higher [58].

The lithic industry at Ambrona features a variety of raw materials: chert, silicified limestone, quartzite, quartz and limestone. All of these except the limestone are allochthonous and were brought to the site by humans [59–60]. The lithic industry found in the Lower Unit is typical of the Acheulean technocomplex [53]. The one in the Middle Unit is characterized by the importance of the flake-production chain and the presence of retouched tools; it has been interpreted as dating from the early Middle Paleolithic [61].

## Methodology

We analyzed the spatial orientation of the archeological remains in order to infer the accumulation processes. This analysis was based on the following stages:

### Compilation of datasets containing published and unpublished data

For Ambrona, we processed two types of spatial datasets: data collected with a total station by the S&PG team from 1993 to 2000, and data collected by the H&F team, available through a few published maps and stratigraphic sections. The H&F team’s maps were made using an archeological grid method. However, there is little published material from the H&F digs, despite the large area that the team excavated during this long period. We were able to fill in this critical gap thanks to Tim D. White, director of the F. Clark Howell Library at the Human Evolution Research Center (HERC), who gave us access to all the unpublished information generated by the H&F team’s work at Ambrona. We scanned 35 dispersion maps and stratigraphic sections into raster files, which enabled us to fill in the puzzle of the Ambrona site.

### Georeferencing spatial datasets in a common coordinate system

We georeferenced all the raster maps and stratigraphic sections retrieved from the H&F team’s documentation, using the coordinate system generated by the S&PG team with a total station. We did this using common points recorded in both datasets, including excavation boundaries, the grid set up for the local coordinate system, and physical reference points left at the site by the H&F dig (trenches, museum buildings, etc). Thanks to these references in the maps, we were able to georeference and record the position of the concentrations of bone remains and lithic artifacts.

### Digitizing maps and stratigraphic sections

**Fig 3:**
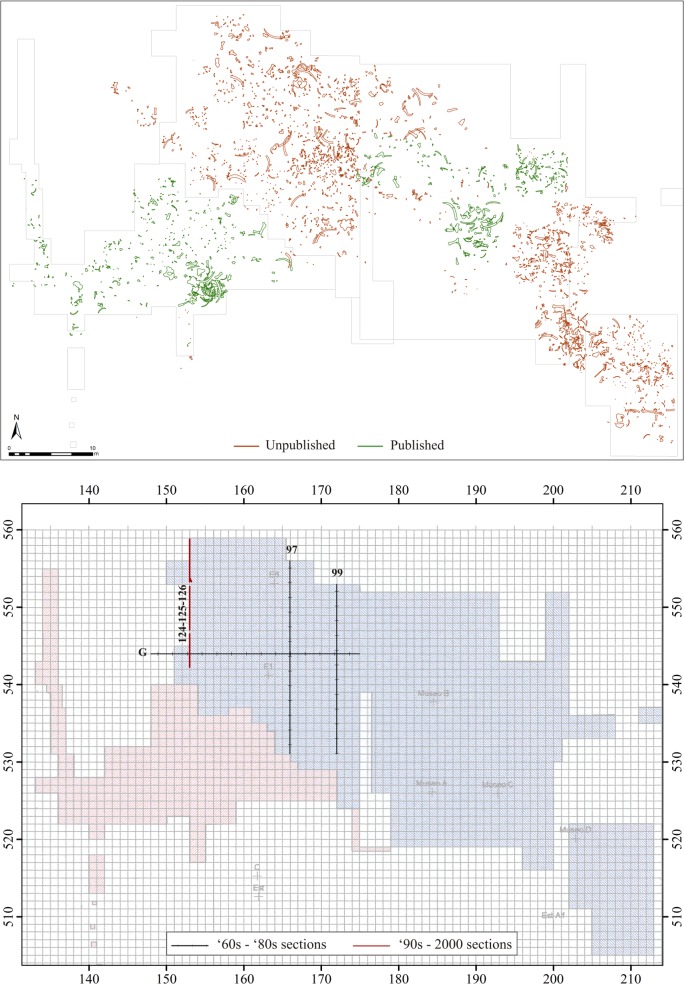
Overall view of the Ambrona site faunal remains. (A) Map of the published and unpublished faunal remains after the maps obtained from the HERC were georeferenced and vectorized; (B) Areas excavated by H&F (blue) and S&PG (red). Location of the sections used to make the stratigraphic correlation.

In this stage, all the archeological remains appearing in the raster maps and stratigraphic sections were vectorized in ArcGIS 10.3. Raster maps were automatically converted into polyline vector files, which were then cleaned up and transformed into polygons [35]. The final result was GIS vector files in which each archeological item was represented by a polygon. A total of 3,019 faunal remains were vectorized from the H&F maps; counting in those already available from the S&PG team’s excavations, we have a total of 4,417 faunal remains from the whole excavated portion of the site (**Fig 3**). Moreover, 3,577 lithic items were likewise vectorized and incorporated in the database. However, since their shape was not recorder on the H&F team’s maps, these objects were represented as point features. We digitized stratigraphic sections made by this team as well, in order to have a reference for comparing these objects’ distribution through the stratigraphic units.

### Correlation of the stratigraphic levels

The maps we retrieved from the F. Clark Howell Library contain no data about the stratigraphic levels where the remains were found. We inferred this information by stratigraphic correlation. We studied the stratigraphic sections made by the H&F team in detail and compared them with those made during S&PG’s fieldwork. The correlation was based on control points common to stratigraphic sections made in different excavation periods. The control points were checked with direct field observations in places where the section were still preserved. Using these control points, the stratigraphic sections and the sedimentological characteristics of the facies were interpolated with the distribution of the stratigraphic units in the area being studied.

### Definition of natural concentrations by means of GIS density maps

We applied statistical tests such as the Chi-square and Kolmogorov-Smirnov to identify the nature of the assemblage. These tests recognize random or clustered distributions, which we mapped by applying K-means and Jenks methods to identify the main concentrations and their locations.

Rcmdr (R-commander) provides useful tools for using K-means, a vector quantization based on the partition of *n* observations into *k* clusters. The observations are divided and reshuffled according to a given criterion, in our case faunal and lithic remains.

All of these tools work with points, which oversimplify the faunal remains data at the working scale. To deal with this limitation, we also applied polygon density methods. Density GIS tools calculate a magnitude-per-unit area from features that fall within a certain neighborhood around each measured point. Only the features that fall within that neighborhood are considered when calculating density. The neighborhoods are calculated using a search radius parameter, which determines the detail of the density surface: high radius values produce a more generalized density raster, while low values produce a more detailed one. In our work we used the Kernel method to generate the density map, which was classified following to the Jenks method (Natural Breaks Classification) provided by ArcGIS 10.3. To produce the final density surface, we tested several search radiuses, and noted that a 2-m value provided good detail, considering the dimensions of the area analyzed and the large size of the items (tusks, pelvis, cranium, mandibles and other large bones). Density analysis was also applied to the lithic items, which were represented as points due to their smaller size. The result of combining faunal and lithic density maps was corroborated by HotSpots analysis, in order to test statistically the spatial correlation between the faunal and lithic remains. HotSpots identifies statistically significant hot spots (high values) and cold spots (low values), grouping all the values with a significant degree of confidence.

### Calculation of orientation data through GIS

The next step was to measure the orientation of the archeological remains with GIS tools. Like the authors of earlier works [28], [37], we used three different methods to estimate the axial direction of each item: 1) the angle of the major axis, or diameter, of a polygon (D), 2) the angle of the minimum bounding rectangle of a polygon (MBR), and 3) the angle of the longest side of a polygon, which is considered the polygon main angle (PMA). These three parameters were calculated for each polygon that represents an archeological item. The study of orientation patterns makes it possible to infer the action of natural processes on anthropogenic assemblages [8], [28], [33–35], [38–39].

### Statistical analysis of orientation data

This analysis concerned faunal remains alone, because very few lithic items were recovered during the S&PG team’s excavations, while those plotted on the maps made by the H&F team were represented only as points. As had been done in earlier works [33–34], we performed orientation analysis only for elongated items (Elongation Index ≥ 1.6) more than 2 cm long, because the positions of items that are round or too small may not reflect orientation processes. Therefore, only 981 of the 3,330 digitized and measured faunal remains were subjected to orientation analysis. They include tusks, a cranium, long bones, pelvis and mandibles. The axial angles of all the items were plotted onto circular histograms, keeping the north at the top as the spatial reference. We used both the Rayleigh and Kuiper tests to differentiate isotropic distribution (uniform) from unimodal, bimodal or multimodal patterns [35], [38–39], [62] and Oriana software for the statistical analysis.

## Results

### Stratigraphic correlation

We studied in detail the stratigraphic sections surveyed by the H&F team, georeferencing their positions using the current coordinate system and taking into account the spatial references indicated in the sections themselves. The stratigraphic sections surveyed by the H&F and S&PG teams were compared in order to identify any coinciding stratigraphic sequences. The H&F team’s sections in the 1960s and 1980s were made according to lithostratigraphic criteria [47], whereas the S&PG team’s sections were made according to allostratigraphic criteria [44]. Lithostratigraphic, sedimentological and thickness parameters were used to compare the two kinds of sections, as well as the presence or absence of faunal remains. The units where we found coinciding control points were AS1 (sands and gravels, fluvial environment), AS3 (clay, lacustrine environment), AS4 (sandy clay and pebbles, fluvial-lacustrine environment), AS5 (sandy-clay and pebbles, fluvial-lacustrine environment) and AS6 (silty clay with sands, fluvial-lacustrine environment).

**Fig 4:**
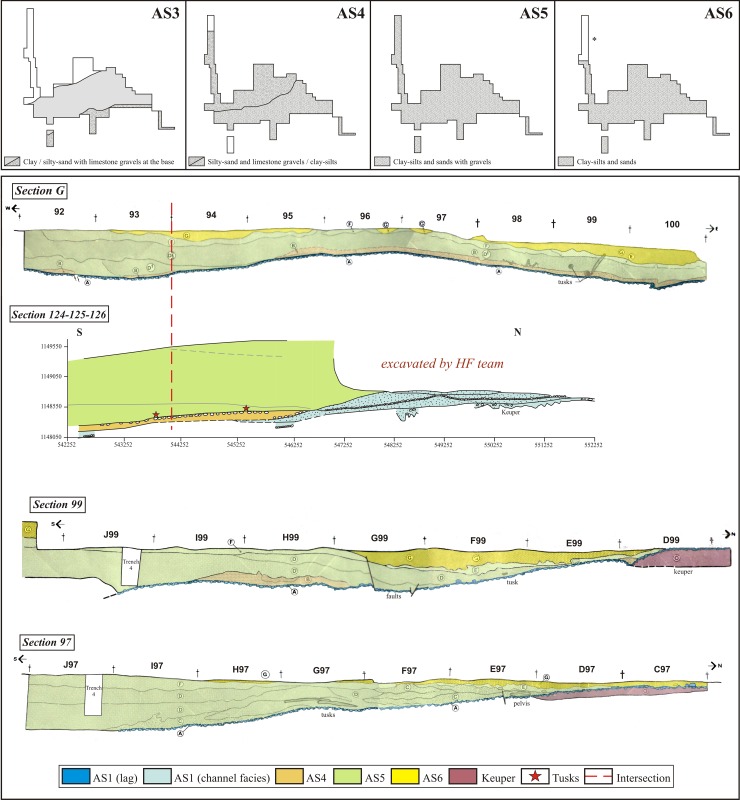
Spatial distribution of the stratigraphic units and correlation between sections. (A) Map showing the limits and extension of the various stratigraphic units at Ambrona (AS3 to AS6); (B) Proposed correlation among the sections made by the H&F team and those made by the S&PG team. (*) AS6 was removed by power shovel and the 1980s fieldwork. The S&PG team did not find this unit in its own fieldwork, so it is not shown in the sections.

The stratigraphic sequence is complete and continuous in the central area of the site, and includes the stratigraphic units from the AS1 lag to the AS6 unit [44]. On the north side, units AS3, AS4 and AS5 display a gradual overlapping geometry, which caused progradation of the palustrine environment. Because of this geometry, the AS3 unit wedges out and disappears towards Y521 (**Fig 4**), so that AS4 rests directly on top of AS1 [44], [48]. Further north (Y545, **Fig 4**), AS4 disappears and AS5 lies on top of AS1. This overlapping explains why similar units cannot be distinguished from one another at the edges of the pond.

Section 124-125-126, made by the current team, crosses section G, which was made in the 1980s. This section shows the thin expression of unit AS4 and its evolution. In the Howell sections, B has a very poor expression and is not present in the northern part of the site. Unit AS4 has two main facies, detritic (north) and clayey-silty (south); it too is absent in the northern part, disappearing towards Y545. At the base of AS5 there is a lag deposit on which lie two tusks. The channel facies of AS1 have a greater development, being the base that AS5 rests directly upon. We correlated levels E, F and D, defined by the H&F team, with unit AS5 (at the base: clay/silt with small percentages of sand and gravels; at the top: clay/mud). The units defined in section G are consistent with the description and the spatial distribution of the AS5 facies.

Unit AS6 was removed by power shovel and in the course of fieldwork carried out before 1989 [44]. That is why this unit is not present throughout the site. What is left of it enabled us to estimate its extension and correlate it with the H&F sections. Section 99 contains evidence of the AS6 deposit and its probable thickness. Unit AS1 is continuous and present throughout the site (**Table 1**).

**Table 1:** Correlation between the units defined by the H&F and S&PG teams.

H&F team	S&PG team
G: Gray, gritty marly loam (north) or clayey mark (south), mottled, strong prismatic structure south.	AS6: Sandy mud and pebbles. Channel and overbank deposits.
E/F: Marly clay/loam, dispersed grits, chaotically bedded (north). Loamy, sandy-texture marls, laminate structure, rich in gastropods (south).	AS5: Mud and silty-sand with pebbles. Channel and overbank deposits.
D: Gritty marly loam, mottling, interdigitating, dispersed pebbles (north), or more homogeneous marly clay.	
C: Gritty marly loam, mottling, prismatic structure, interdigitating, dispersed pebbles (north) or more homogeneous marly clay.	
B: Semi-stratified, pebbly / gritty / marly clay.	AS4: Silty clay and limestone gravels / clay-silt. Channel and overbank deposits.
A: Strat. med. subrounded gravel in calc. clay matrix. Some microfaults.	AS1: Sands and limestone gravels. Fluvial environment.

The **Fig 4** shows the boundaries and extension of the deposits: AS3 is limited to the central and NE part of the site, whereas units AS4 and AS5 widen towards the edges of the site. The expansion of units AS4 and AS5 was probably due to the increase in river discharge from the NE basin, which caused progradation of the lake shores. The information provided by the H&F stratigraphic sections shows a considerable presence of lithic items and faunal remains in units AS3, AS4 and, to a lesser extent, AS5; this indicates that all the Ambrona stratigraphic units contain macrofaunal remains. The maps made by the H&F team show a very low presence of lithic industry, which is consistent with the characteristics of the deposit and the possible selection of materials by size. Thanks to the information provided by these stratigraphic sections, by the lithological descriptions and by the faunal and lithic assemblages found, we were able to reconstruct the distribution of the Ambrona stratigraphic units, which we then combined with the orientation patterns to assess the accumulation processes pertaining to each natural concentration.

### Density analysis

**Fig 5:**
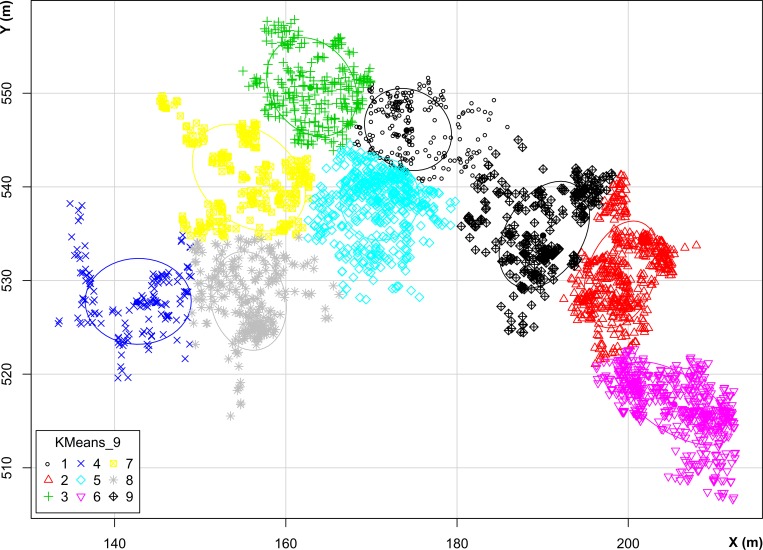
K-means distribution map. Map of the distribution of materials and their classification according to the k-means method.

The results obtained from the Chi-square and Kolmogorov-Smirnov (K-S) tests point to clustered distributions. The chi-square value is higher than the critical value (3,693E+21 > 35,172), hence the distribution is statistically different. The observed distribution is clustered, not random. On the other hand, K-S shows that the largest absolute difference is higher than the critical value (0,398 > 0,021), hence the gap between observed and expected values can be considered statistically different. The null hypothesis of randomly distribution of points is rejected. Because these statistical tests showed clustered distributions, we used k-means method to identify the clusters (**Fig 5**).

The K-means method groups data according to the nearest mean, so it classifies the whole distribution into various clusters; it does not distinguish between larger and smaller assemblages. Accordingly, we made polyline density maps to identify de main concentrations and their intensity, taking into account the shape and size of the items.

**Fig 6:**
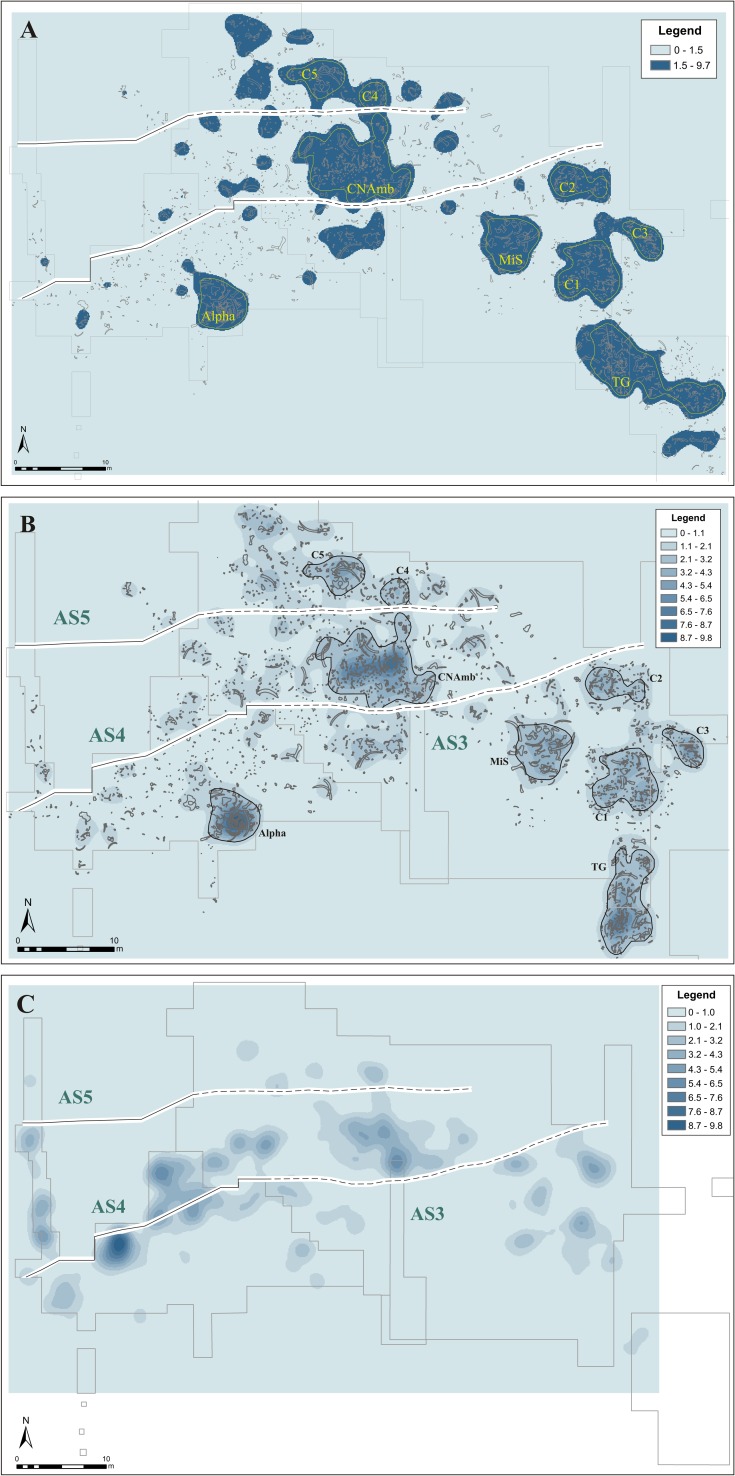
Density maps of the faunal and lithic remains, obtained with Kernel and Jenks algorithm. The lines mark off the extension of the stratigraphic units (for more information about the limits of the units, see Fig 4). The largest accumulation of lithic items is located in the “belt” containing the AS4 unit.

The bone density map, which was made with a search radius of 2 m, shows a mean value of 4.33–5.4 items per square meter, a maximum of 9.46 items and a minimum of 1. The spatial distribution of these values is heterogeneous, with a low background value of around 0–1.08 and density peaks of 9.72–9.76 items per square meter. The Kernel density maps were classified using the Jenks method (**Fig 6**), in order to separate the highest concentrations from the lower-density areas. We then selected the concentrations located in specific stratigraphic contexts and containing enough items to enable us to perform statistical orientation analysis.

Based on this approach, we suggest that there are three main concentrations of faunal remains in the excavated area: one in the south (Alpha), one in the central-northern part (CNAmb) and one in the west (TG).

The Alpha concentration is defined by densities of between 2.26–9.72 bones per square meter (**Fig 6**), referring to an almost complete elephant skeleton (*Palaeoloxodon antiquus*) excavated in 1995 [52], [63]. Most of the skeletal parts are preserved, some of them in anatomical connection. Taphonomic studies suggest that the original position of the bones was slightly altered by natural processes and by animals (trampling, diagenetic alterations, scavenging), but we do not rule out the possibility of human intervention. The lithic items were found on the periphery of the accumulation, and the finds indicate that they had been deposited at the same time as the sediments [63]. The map shows a large accumulation of remains in a specific area, with no wide dispersion.

The CNAmb concentration is located in an area that was excavated in 1980. This assemblage displays a wider dispersion (**Fig 6**), and includes several tusks around 3 m long and a large amount of small-sized remains (the smallest ones are 5–10 cm). At first, the stratigraphic unit that contains this accumulation was unknown, since there is no reference to it in the maps or in the written documentation. However, the overlapping geometry observed when we performed the stratigraphic correlation suggests that this concentration could belong to the AS4 unit.

The TG concentration was published in 1963 in the *Noticiario Arqueológico Hispánico* [49]. This part of the site was excavated in 1962, and the sole stratigraphic reference to it appeared on the map, where the elephant bones were shown in different colors and symbols to indicate which level (Upper, Middle or Lower) they had been found in. However, these locations seem to indicate the bones’ spatial position, because they do not reflect any previously defined stratigraphic unit. The stratigraphic correlation performed in our study suggests that this concentration might belong to AS3, since this unit is dominant in this area because of its position and thickness. Moreover, the dispersion and size of the remains are very similar to those of the other accumulations we found in AS3. As the density map shows (**Fig 6**), the bones are less concentrated in TG than in Alpha. There is also a prevalence of long and large bones in TG, unlike the situation in the CNAmb concentration.

Besides these three main concentrations, other ones have been identified in unit AS3. The characteristics of concentrations C1, C2 and C3 are more similar to those of TG, where there are smaller remains and the assemblage does not consist solely of large bones. In the northern part of the site we found other two concentrations–C4 and C5 –likewise ascribed to AS5. The boundary of AS5 is very clear, which is why we could correlate these concentrations to this unit. These assemblages are made up of only a few items. The largest are a pelvis and a tusk, both found in C5.

To produce the lithic density map, we likewise used a search radius of 2 m, which shows a mean value of 4.35–5.4 items per square meter, a maximum of 9.8 items and a minimum of 1. The spatial distribution of these values is heterogeneous, but we observe a continuous area where lithic density is concentrated. The main concentration is defined by densities between 1 and 9.8 items per square meter, the other by densities between 2.08 and 6.5 items per square meter. However, all the concentrations show very little presence of industry: the density of lithic items is very low compared with the density of bone remains.

Two main concentrations have been identified: one is located in the south-west part of the site and is very compact; the other, more dispersed, is in the central-eastern part and almost coincides with the CNAmb concentration (**Fig 6**). These two concentrations are part of a “belt” containing most of the lithic accumulations. The largest concentration of lithic remains coincides with the boundaries of unit AS4, which locally erodes AS3 and yielded 353 pieces [53]. The unit that contains the largest number of lithic items is AS4; the other units also contain accumulations of faunal remains but hardly any lithics.

The *chaîne opératoire* phases identified in the artifacts found during the 1993–2000 digs provided some information. The assemblage contains elements of all sizes, but they were found in derivative, not autochthonous, positions [53]. Unit AS3, where we found a considerable amount of bones and an almost complete elephant skeleton, contained only 74 lithic pieces in a 412 m^2^ area. The main lithic artifacts found here are flakes in the final production phase. In this unit, deposit accumulation mechanisms include small channel facies that may have brought external elements from the vicinity into the pond [53].

In unit AS5, the contrast between the quantity of lithics and the quantity of bones is even sharper: only 5 lithic items were recorded in the 17.5 m^2^ preserved [53]. These data refer to the excavations carried out by the S&PG team; industry finds were rarely recorded on the excavation maps produced by the H&F team. Access to the H&F team’s documentation, however, has enabled us to figure out the position and density of the lithic finds. The information we obtained by analyzing the facies and the rest of the lithic finds can be extrapolated to the H&F team’s maps, because the reported distribution of the pieces coincides with the description of each unit made by the current team [44], [53]. There are hardly any lithic items in the northern part of the site, where AS5 presumably lies, but their number increases in the area where unit AS4 is.

### Orientation patterns

**Fig 7:**
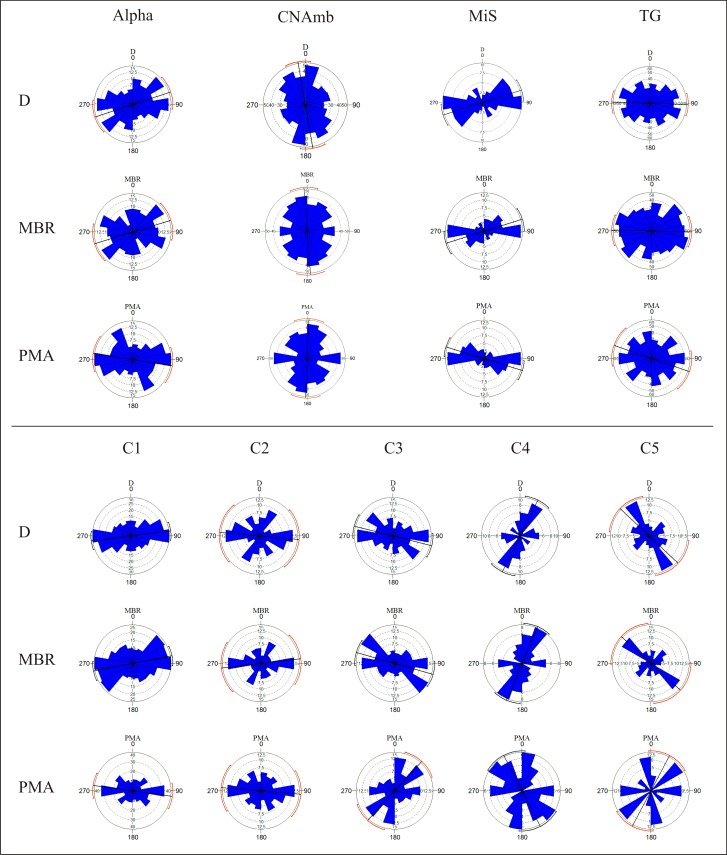
Circular histograms produced using the D, MBR and PMA methods and showing the distribution of the various accumulations of bones (only items with elongation index > 1.6 and size > 2 cm).

Circular histograms show the different orientation patterns of the Ambrona assemblages (**Fig 7**). The Alpha concentration produces mainly dispersed histograms but with a certain trend to E-W directions (**Fig 7**). However, the high *p* values resulting from the Rayleigh and the Kuiper tests (**Table 2**), when checked against unimodal, bimodal or multimodal distributions, do not provide any evidence of a departure from uniformity. C2’s circular histogram shows a similar E-W dispersion trend (**Fig 7**), and the Rayleigh and Kuiper tests did not detect any evidence of preferred orientations. Therefore, neither Alpha nor C2 show any strong evidence of departure from uniformity, thus supporting the conclusion that fossils in these concentrations are oriented at random.

**Table 2:** Dispersion parameters and statistical test of the items found at Ambrona.

		size	Vector (°)	Mean Vector		Deviation (°)				
		*n*	*μ*	*r*	*k*	*v*	*Z*	*p*	*V*	*p*
**Alpha**	*D*	83	71.57	0.17	0.35	53.68	2.48	0.084	1.470	> 0.15
** **	*MBR*	83	72.85	0.15	0.30	55.87	1.85	0.157	1.292	> 0.15
** **	*PMA*	83	98.78	0.17	0.34	54.38	2.26	0.104	1.562	0.15 > p > 0.10
**CNAmb**	*D*	290	169.99	0.14	0.28	56.96	5.57	0.004	2.386	< 0.01
** **	*MBR*	290	175.53	0.13	0.25	58.36	4.57	0.010	2.018	< 0.01
** **	*PMA*	290	2.50	0.11	0.22	60.45	3.38	0.034	1.954	< 0.025
**TG**	*D*	332	92.06	0.13	0.26	58.10	5.43	4.00E-03	2.132	< 0.01
** **	*MBR*	332	91.13	0.10	0.19	61.92	3.11	4.50E-02	1.778	< 0.05
** **	*PMA*	332	109.27	0.07	0.14	65.77	1.71	1.81E-01	1.613	0.15 > p > 0.10
**MiS**	*D*	42	72.32	0.38	0.81	40.02	5.97	0.002	2.400	< 0.01
** **	*MBR*	42	72.58	0.34	0.73	41.87	4.96	0.006	2.122	< 0.01
** **	*PMA*	42	107.23	0.41	0.90	38.26	7.06	6.83E-04	2.276	< 0.01
**C1**	*D*	143	83.09	0.25	0.52	47.68	8.96	1.28E-04	2.497	< 0.01
** **	*MBR*	143	78.85	0.23	0.48	49.00	7.67	4.68E-04	2.498	< 0.01
** **	*PMA*	143	97.88	0.18	0.37	53.03	4.65	0.01	2.113	< 0.01
**C2**	*D*	55	95.10	0.12	0.24	59.40	0.75	0.474	1.169	> 0.15
** **	*MBR*	55	83.25	0.14	0.28	56.86	1.07	0.343	1.365	> 0.15
** **	*PMA*	55	94.78	0.15	0.31	55.63	1.27	0.282	1.320	> 0.15
**C3**	*D*	67	104.25	0.27	0.56	46.48	4.82	0.008	1.955	< 0.025
** **	*MBR*	67	105.85	0.25	0.52	47.68	4.20	0.015	1.959	< 0.025
** **	*PMA*	67	42.54	0.18	0.36	53.45	2.06	0.127	1.797	< 0.05
**C4**	*D*	33	26.62	0.35	0.75	41.48	4.05	0.016	1.761	< 0.05
** **	*MBR*	33	22.92	0.33	0.71	42.44	3.68	0.024	1.782	< 0.05
** **	*PMA*	33	152.77	0.22	0.44	50.17	1.54	0.216	1.636	0.10 > p > 0.05
**C5**	*D*	46	133.33	0.16	0.32	55.25	1.12	0.33	1.399	> 0.15
** **	*MBR*	46	132.10	0.13	0.27	57.52	0.82	0.444	1.559	0.15 > p > 0.10
** **	*PMA*	46	27.80	0.19	0.38	52.48	1.60	0.202	2.490	< 0.01

Conversely, the circular histograms of the C1 concentration show a clear unimodal pattern highly clustered around the E-W direction. The lowest *p* values strongly reject uniformity for this assemblage, for they indicate a dominant unimodal distribution (**Table 2**). This situation is similar to the one in the MiS concentration, where circular histograms (**Fig 7**) show a principal E-W mode and the *p* values obtained with the Rayleigh tests are < 0.002 (**Table 2**). These data provide very solid evidence of a preferred E-W unimodal orientation for all the measurements considered (D, MBR and PMA).

In the C3 accumulation, D and MBR circular histograms show a similar trend, a mean ENE-WSW direction (**Fig 7**), whereas PMA histograms show a main perpendicular mode (NE-SW) and minor E-W modes. Statistical tests give clear evidence of preferred unimodal orientation for D and MBR measurements (Rayleigh *p* values < 0.015, **Table 2**), while PMA measurements likewise suggest some evidence of preferred orientation, though it leans more toward bimodal distribution (Kuiper *p* value = 0.05).

Orientation patterns in the northern part of the site suggest several trends (N-S, E-W, NNE-SSW and NW-SW). The CNAmb and TG concentrations tend to show circular distributions dominated by a unimodal direction: N-S in the CNAmb and E-W in the TG (**Fig 7**). In both concentrations, minor modes are not significant except for PMA measurements, where they become more representative. Statistical tests show very low *p* values for the CNAmb and TG concentrations (**Table 2**), which makes it possible to reject uniformity. In C4 and C5, the circular histograms show clear trends in the D and MBR measurements (**Fig 7**), and high dispersion for PMA. In C4, the D and MBR measurements show a main NNE-SSW direction and a minor E-W mode. C5 displays a major NW-SE trend and minor modes around the NE-SW directions. For PMA the pattern is very dispersed, with no dominant trends. Regarding the statistical tests, C4 provides solid evidences of departure from uniformity. Conversely, in C5 the low *Z*, *V* values and the high *p* values do not provide clear evidence of any preferred orientation of fossils (**Table 2**). Thus, though C5’s circular diagram shows dominant orientations, we did not find any statistical significance in it.

**Fig 8:**
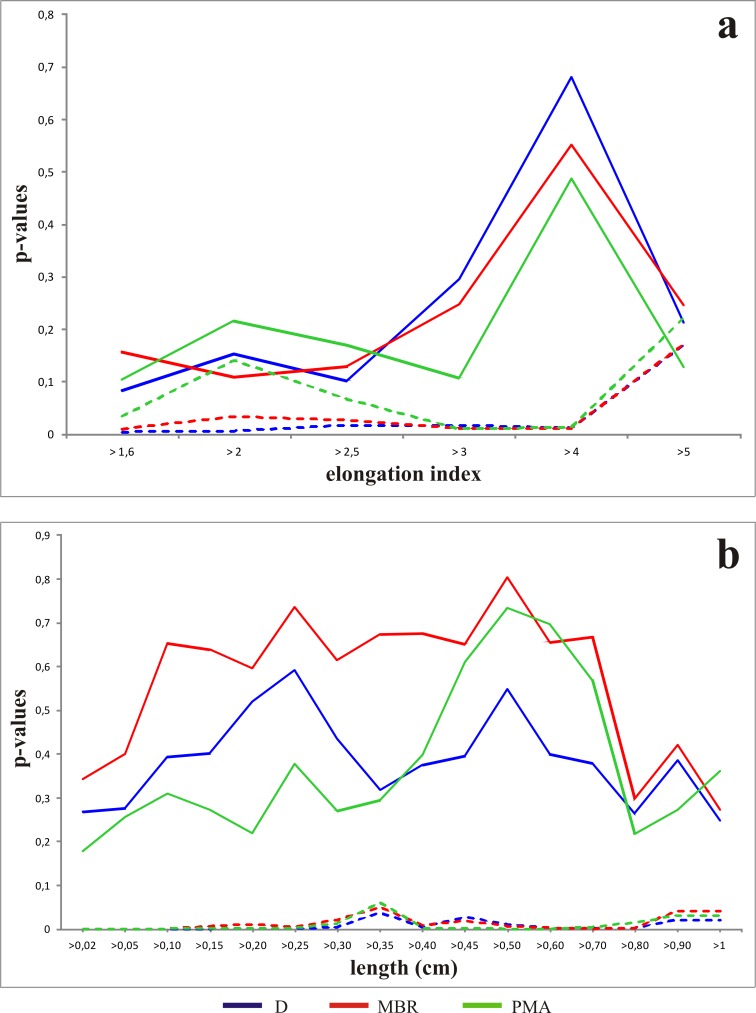
**Variation of *p*-values according to the elongation index (a) and length (b) of the faunal remains.** The solid line delimitates the Alpha concentration, the broken line indicates the CNAmb concentration.

To assess the influence of the lengths, shapes and sample sizes (*n*) of the items on the statistical significance of the analysis, we selected the Alpha and CNAmb concentrations for our analysis of the evolution of the *p* values according to these variables. **Fig 8** shows the evolution of the *p* values calculated for the two larger assemblages (Alpha and CNAmb), using D, MBR and PMA angular measures. We chose these assemblages for this comparison because their orientation patterns differ and their sample size is larger. *P* values were calculated according to the elongation index and sample size *n* (or number of observations). The evolution of the *p* values according to the elongation index shows a wide gap between the two larger assemblages (**Fig 8A**). The Alpha assemblage always has high *p* values (close to or higher than 0.1), which indicates that a hypothesis of randomness for this concentration’s orientation cannot be rejected, regardless of variation in the elongation index. The *p* values of CNAmb, however, are mostly lower than 0.05; the hypothesis of a random distribution of orientations may thus be confidently rejected, with no significant influence of the variation of the items’ elongation. As regards changes in *p* values depending on the length of the items (**Fig 8B**), the difference between the two concentrations is likewise clear. Alpha’s *p* values, computed from D, MBR and PMA measurements, are always higher than 0.15, which by the same token suggests that there is no evidence of departure from uniformity, in this case regardless of length. On the other hand, CNAmb’s *p* values are very low and close to 0, which indicates that a very significant departure from uniformity occurs in conjunction with variations in the items’ length.

## Discussion

**Fig 9:**
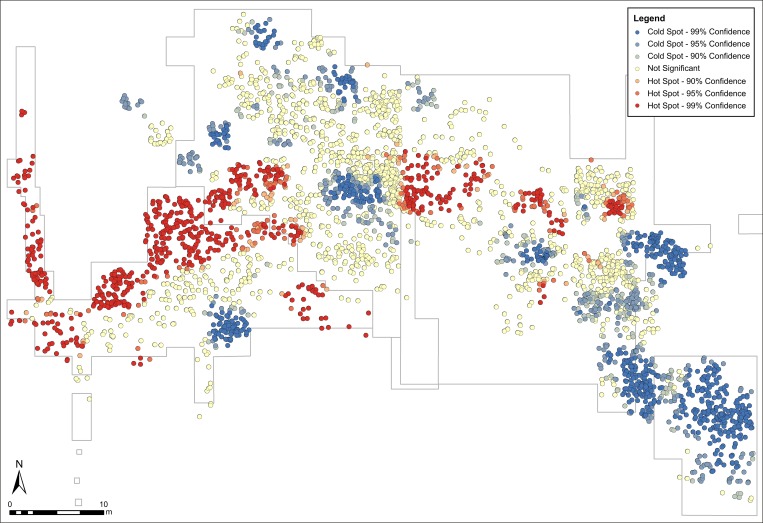
HotSpots map. The map shows the layout of the concentrations of faunal and lithic remains which have a higher confidence value referred to their spatial location.

Density maps show partial coincidence among the main concentrations of fauna and industry. The latter is evident in the areas defined by the CNAmb, C2, C4 and, to a lesser degree, C1 concentrations (**Fig 6**). As **Fig 9** shows, the location of the main assemblages highlighted by HotSpots coincides with the data provided by density maps. The lake shoreline is where we found the greatest concentration of materials, both faunal and lithic.

However, in other areas, such as Alpha, the concentrations of faunal remains and of lithic items do not coincide. This lack of coincidence between these concentrations is related to the stratigraphic unit and the paleogeographic position in which they are found. The Alpha concentration is located in the central parts of the AS3 unit, where very few lithic items have been recorded [53]. The lithic industry is located around, rather than within, the concentrations of faunal remains [63]. Conversely, lithic artifacts are more abundant in the AS4 unit [53] and in the peripheral areas of AS3 (**Fig 6**). This distribution could be due to 1) human activities on the shores of the wetland (where we found several lithic items); or 2) natural processes (overland flow, water level fluctuations, waves) that concentrated the remains in those areas. These two possibilities were assessed by means of orientation analysis.

Most of the concentrations described are defined by a very clear preferred orientation pattern. CNAmb, TG, MiS, C1, C3 and C4 display preferred orientations, which are statistically very significant and are dominated mostly by unimodal distributions (**Fig 7** and **Table 1**); this suggests that natural processes oriented the items. On the contrary, uniformity distribution cannot be rejected for the C5, C2 and Alpha concentrations, hence we suggest that they are random accumulations resulting from non-orientation natural processes.

**Fig 10:**
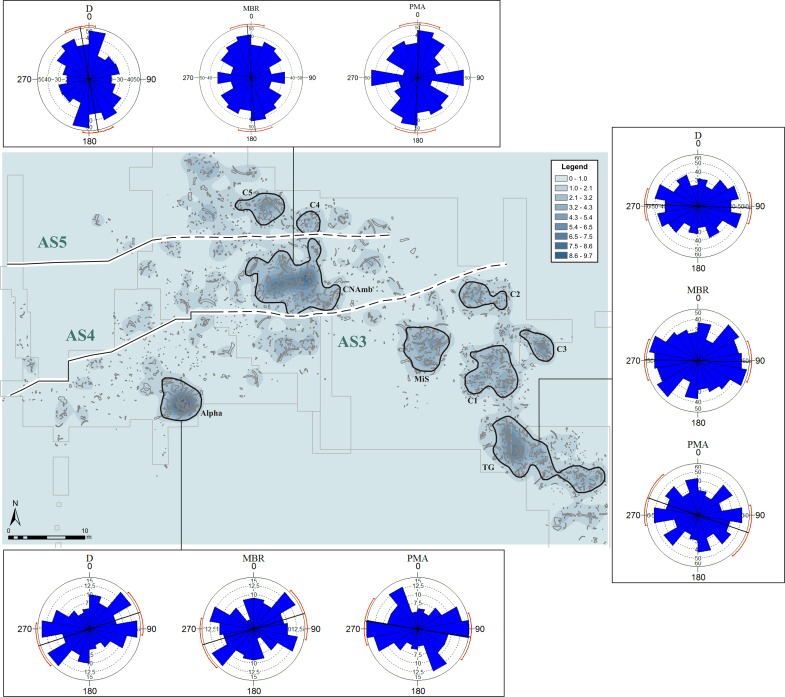
Density and map of faunal remains distribution. Circular histograms of the main concentrations show the trend of the assemblages according to their location.

Furthermore, the orientation distribution shows spatial patterns that depend on the site zone (**Fig 10**). Orientation patterns with a predominant E-W trend are located in the eastern part of the site and pertain to the AS3 unit (MiS, C1, C3 and TG concentrations). Circular histograms for Alpha and C2 (in the southern part of the site) are similar to this first group, although statistical tests do not confirm preferred orientation. On the other hand, a third group of orientation patterns is located toward to the northern part of the site, displaying NNE-SSW, NW-SE, and minor E-W directions (C4 and C5 concentrations developed in unit AS5). The CNAmb concentration would belong to this third group, which occurs in a different stratigraphic unit (AS4) and is characterized by a N-S pattern. This pattern differs from those in the other histograms, but may resemble those in the C5 and C4 concentrations in unit AS5.

The association between orientation patterns and stratigraphic units points to a correlation between the accumulation processes that produced the concentrations and the sedimentological characteristics of the units. Orientation patterns can thus be classified into three groups that reflect different stratigraphic units and inputs.

The first group consists of the concentrations located in the northern part of the site. These are defined mainly by NNE-SSW and NW-SE directions, which might have resulted from flow inputs from the other valley that fed the Ambrona wetland from the north-east [44]. Flows coming from this direction could have determined a NE-SW orientation of the items–that is, aligned them parallel to the flow–or a NW-SE orientation, if the elongated items rolled on the surface and halted perpendicularly to the direction of the flow [35]. This would explain the main directional components of the orientation patterns observed in concentrations C4 and C5 in unit AS4, which contains water and mud-flow facies. They suggest that water inputs into this part of the site came from the valley situated north-east of the site.

The second group of orientation patterns is seen in the CNAmb concentration, which is likewise assigned to the AS4 stratigraphic unit. CNAmb displays a main N-S directional mode that suggests two possible causes: 1) inflows from the NE valley, with small variations in channel direction, due perhaps to the sinuous nature of the streams; and 2) inflows from the eastern valley. In the latter case, most of the items would have rolled perpendicularly to the flow, while some would have assumed an E-W orientation parallel to the flow, as MBR and PMA circular histograms seem to show (**Fig 7**). Based on stratigraphic observation of this unit, in places where mud channels have been documented as having a NE direction and AS4 contains sandy-clay and pebbles (**Fig 4A**) [44], the first possibility would seem the more likely one. From a spatial point of view, oriented concentrations are located mainly along the edges of the site, closest to the lateral valleys.

The third group consists of the concentrations found in AS3, which tend to display an E-W direction (**Fig 7**). This direction coincides with that of the inflow from the other main valley that fed the Ambrona wetland area [44], which runs from east to west. Accordingly, the orientation of these faunal concentrations may have resulted from E-W flows coming mainly from this valley. However, no significant evidence of water current facies or mud channels was documented in AS3. Only small channel facies were observed; it has been suggested that they may have transported lithics from the vicinity into the pond [53]. Orientation processes may have been induced by these channels, though bone orientation may also have originated in an intra-basin environment–that is, in the pond–where waves and water level variations would have prevented water current facies from entering the pond.

Regarding the concentrations with random orientation distribution, C5 and C2 are surrounded by oriented concentrations, so they are less clear. The Alpha concentration, however, is isolated from others that display a preferred orientation. It is located in the central part of the preserved site, where the central facies of AS3 has been identified [44], and it was probably less affected by inflows from the neighboring valleys. The absence of any preferred orientation in the Alpha concentration suggests that it was not reworked by erosion and/or transportation by geological processes. Taphonomic studies show that some of the bones were in anatomic position, others in anatomic proximity [63]. The positions of six ribs and a thoracic vertebra indicate that they had been displaced because of the plasticity of the mud in which they lay. Animals seem not to have caused extensive changes in the bones’ position (by trampling, scavenging, etc.) because we found a great number of vertebrae and ribs. These bones are the most fragile of all, and become unrecognizable if subjected to intense trampling. Moreover, in marshland, continuous trampling would have caused the ribs to be rapidly buried [64]. In the case of the Alpha concentration, we did not note any differences in the positions of these remains; they all lay rather flat. The positions of the other bones (scapulas, tusks, etc.) suggest that their displacement could have been caused by trampling and manipulation by other elephants [63]. Moreover, the bones are fissured, which may indicate that alternating humid and dry conditions caused them to expand and contract. Hence, the area where the Alpha concentration is located seems to have undergone cycles of dampness and dryness [63]. The orientation patterns show that there is no preferred direction; therefore, the concentration may have been exposed to erosion and transporting processes, but not for long.

The MiS concentration, which is located in unit AS3, shows signs of having been exposed over a longer period of time than the Alpha concentration. Taphonomic studies determined that the MiS assemblages contain 4 or 5 individuals, but only 42 bones. These data point to processes that could have occurred above this assemblage. Its higher position would not have furthered preservation of the bones. MiS would have been more exposed to post-depositional processes. Preliminary studies of this concentration suggest that water was the agent that had determined the orientation of the bones; other factors, such as displacements in the paleosoil, were ruled out. One tusk was fractured, which might have been due to some deformation of the paleosoil that occurred after the remains were buried. The bones tilt 40 cm to the north. The taphonomic data therefore support the findings of the orientation pattern analysis, confirming than the AS3 concentrations with preferred orientations, such as MiS, were more affected by post-depositional factors than was the Alpha concentration, which does not display any preferred orientation pattern.

The analysis performed to assess the consistency of orientation patterns in Alpha and CNAmb indicates that their orientation patterns are indeed consistent regardless of the items’ shapes and sizes. Where orientation patterns do show a preferred orientation, our study suggests that processes which caused preferred orientations would have sufficed to orient large items, some of them up to 3 m long.

Integration of the new planimetries and the correlations among stratigraphic sections indicate that the level containing a larger quantity of lithic items corresponds to the detritic facies of the AS4 unit. On the other hand, decantation facies (clays) like those in unit AS3 contain hardly any lithics. This pattern also applies to the dispersion of faunal remains. The maps show that in some areas small-sized remains predominate, while others contain more long and large bones. CNAmb contains a larger accumulation of small-sized remains, and its orientation patterns point to the existence of preferred orientations of the fossils; this is consistent with the characteristics of unit AS4.

Unit AS5 contains fewer items, though some faunal and lithic remains were found there. *Palaeoloxodon antiquus* remains (tusks) were identified in the H&F team’s sections and in those being excavated now. Hardly any were found during the 1990s and 2000s because most of the levels had already been excavated by the H&F team. It is possible that the remains currently associated with unit AS5 were actually reworked from units AS4 or AS1 (depending on the position). The characteristics of the deposit, the total amount of excavated remains, their characteristics and their orientation patterns indicate that the deposit is not a rich one, but neither is it sterile. At present, we cannot determine whether the remains in AS5 were reworked from the subjacent level or were transported to the site by the inflow from the basin lying to the north.

## Conclusions

For the first time, all the available information from the Ambrona site, gained through the excavation of over 3,000 m^2^, has been unified. Access to all the documentation generated by the Howell and Freeman team (1962–1963, 1980, 1981 and 1983) has made it possible to correlate the investigations carried out by two different teams with different stratigraphic interpretations of this site. Georeferencing all the maps of the excavated materials and the stratigraphic sections, correlating the sections interpreted in the 1960s and 1980s with the current stratigraphy, and making a complete spatial study of the assemblages have enabled us to have a more accurate and integrated view of the formation processes that occurred at Ambrona.

Our density and orientation analyses of these maps constitute an initial contribution to the study of the accumulation processes that affected the concentrations of large mammal remains that characterize the Ambrona site. We are now able to define the main concentrations present at Ambrona, which display different orientation patterns, depending on which stratigraphic unit they are in. This circumstance suggests that accumulation patterns are closely linked to the stratigraphic units. Orientation analyses show that most of the concentrations display preferred orientation patterns. Exposure to and orientation by natural processes may have been more intense for concentrations located in shore areas of the wetland (CNAmb, TG, MiS, C1, C3, C4). One concentration was also identified in a more central part of this wetland; its random orientation pattern indicates that it did not undergo any orientation process during its accumulation.

The Alpha assemblage consists of a nearly whole elephant skeleton. As it was located in the deeper part of the pond, the bones were quickly buried and preserved by rapid sedimentation. This is consistent with the data obtained by available taphonomic studies, which show that oriented accumulations had been more subjected to post-depositional processes than those with random orientation patterns.
